# PMAP-36 reduces the innate immune response induced by *Bordetella bronchiseptica*-derived outer membrane vesicles

**DOI:** 10.1016/j.crmicr.2020.100010

**Published:** 2020-09-25

**Authors:** Melanie D. Balhuizen, Chantal M. Versluis, Roel M. van Harten, Eline F. de Jonge, Jos F. Brouwers, Chris H.A. van de Lest, Edwin J.A. Veldhuizen, Jan Tommassen, Henk P. Haagsman

**Affiliations:** a Section of Molecular Host Defence, Division of Infectious Diseases and Immunology, Department of Biomolecular Health Sciences, Faculty of Veterinary Medicine, Utrecht University, Yalelaan 1, 3584 CL, Utrecht, The Netherlands; b Section Molecular Microbiology, Department of Biology, Faculty of Science, Utrecht University, Utrecht, The Netherlands; c Center for Molecular Medicine, University Medical Center Utrecht, Utrecht, The Netherlands; d Section of Cell biology, Metabolism and Cancer, Division of Infectious Diseases and Immunology, Department of Biomolecular Health Sciences, Faculty of Veterinary Medicine, Utrecht University, Utrecht, The Netherlands

**Keywords:** *Bordetella bronchiseptica*, Outer membrane vesicles, Host defense peptides, Cathelicidins, PMAP-36, Vaccine development

## Abstract

Host defense peptides (HDPs), such as cathelicidins, are small, cationic, amphipathic peptides and represent an important part of the innate immune system. Most cathelicidins, including the porcine PMAP-36, are membrane active and disrupt the bacterial membrane. For example, a chicken cathelicidin, CATH-2, has been previously shown to disrupt both *Escherichia coli* membranes and to release, at sub-lethal concentrations, outer membrane vesicles (OMVs). Since OMVs are considered promising vaccine candidates, we sought to investigate the effect of sub-bactericidal concentrations of PMAP-36 on both OMV release by a porcine strain of *Bordetella bronchiseptica* and on the modulation of immune responses to OMVs. PMAP-36 treatment of bacteria resulted in a slight increase in OMV release. The characteristics of PMAP-36-induced OMVs were compared with those of spontaneously released OMVs and OMVs induced by heat treatment. The stability of both PMAP-36- and heat-induced OMVs was decreased compared to spontaneous OMVs, as shown by dynamic light scattering. Furthermore, treatment of bacteria with PMAP-36 or heat resulted in an increase in negatively charged phospholipids in the resulting OMVs. A large increase in lysophospholipid content was observed in heat-induced OMVs, which was at least partially due to the activity of the outer-membrane phospholipase A (OMPLA). Although PMAP-36 was detected in OMVs isolated from PMAP-36-treated bacteria, the immune response of porcine bone-marrow-derived macrophages to these OMVs was similar as those against spontaneous or heat-induced OMVs. Therefore, the effect of PMAP-36 addition after OMV isolation was investigated. This did decrease cytokine expression of OMV-stimulated macrophages. These results indicate that PMAP-36 is a promising molecule to attenuate undesirable immune responses, for instance in vaccines.

## Highlights

• Sub-lethal PMAP-36 treatment of bacteria increases outer membrane vesicle release.
• Lipidomic analysis revealed the OMV lipidome upon PMAP-36 or heat treatment.
• Supplementation with PMAP-36 attenuated undesirable OMV-induced immune responses.

## Graphical abstract

**Figure:**
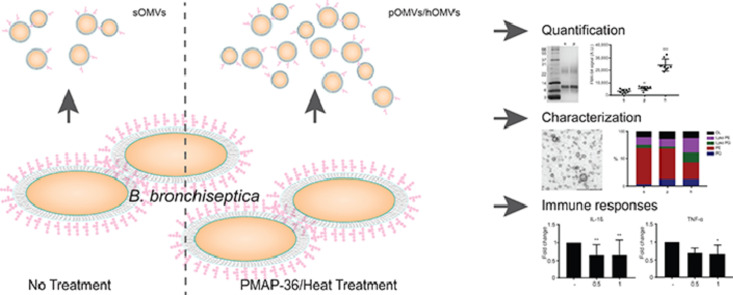

## 1 Introduction

Outer membrane vesicles (OMVs) are spherical particles, 20-300 nm in size, that are naturally produced by all Gram-negative bacteria (Schwechheimer and Kuehn, 2015, Deatherage et al., 2009). OMVs represent the outer membrane (OM) of the Gram-negative bacterium and comprise a large number and wide variety of surface-exposed antigens. This makes OMVs promising in vaccine development as has already been shown for *Neisseria meningitidis* and *Bordetella pertussis* (Holst et al., 2013, Holst et al., 2009, Granoff, 2010, Keiser et al., 2011, van de Waterbeemd et al., 2013, Raeven et al., 2016, Raeven et al., 2015, Gaillard et al., 2014, Roberts et al., 2008, Hozbor, 2017). Furthermore, the lipopolysaccharide (LPS) of OMVs acts as endogenous adjuvant, which is an additional advantage. However, large amounts of LPS can also cause the host immune system to overreact, evoking adverse effects against vaccine formulations. Injection with *E. coli* LPS has been shown to increase body temperature and heart rate, as well as white blood cell counts in healthy human volunteers (Copeland et al., 2005). Currently, LPS can be removed by detergent treatment, but thereby also important antigens, such as lipoproteins, are removed. Furthermore, production of large quantities of spontaneously released OMVs (sOMVs) is challenging. Current methods to induce OMV release, e.g. genetic modification and/or isolation through detergent treatment, may significantly alter OMV properties. A method to induce OMV release and maintain a native composition could be exposure of bacteria to natural stress, for instance heat (von Bergen et al., 2012, Eberlein et al., 2018) (accompanying manuscript by E. F. de Jonge) or Host Defense Peptides (HDPs).

HDPs are small, cationic, amphipathic peptides, naturally found in many species. They are produced by several types of cells, such as neutrophils and epithelial cells, and form an important part of the innate immune system (Hazlett and Wu, 2011, Zasloff, 2002, Zanetti, 2004, van Harten et al., 2018, Mookherjee et al., 2020). One class of HDPs, cathelicidins, includes the porcine PMAP-36. This HDP is α-helical with a hinge region at the C-terminus containing a cysteine residue which allows the peptide to dimerize. These dimers are vital for the peptide's ability to interact with and neutralize LPS (Scheenstra et al., 2019). Thus far, several models for the interaction between HDPs and bacterial membranes have been proposed, all leading to membrane permeabilization and cell death (Zhang and Gallo, 2019).

Since HDPs attack the bacterial membrane, their mechanism is hypothesized to induce OMVs. OMV release is known to increase as Gram-negative bacteria respond to stressors. For instance, OMV release is increased when misfolded outer-membrane proteins accumulate in the periplasm through induction of the σ^E^ stress response (Schwechheimer and Kuehn, 2013). Additionally, OMV release is upregulated by environmental stress, such as antibiotic exposure (McBroom et al., 2006, Chan et al., 2017, Devos et al., 2017). However, while vesicle release of *E. coli* has been observed at sub-bactericidal concentrations of CATH-2 (Schneider et al., 2016), the effect of HDPs on OMV release has yet to be quantified.

In this study, we investigated the effects of sublethal concentrations of a very potent porcine HDP, PMAP-36, on OMV release in a porcine, pathogen *B. bronchiseptica*, with the goal to increase release of OMVs that are suited for vaccine usage. We compared PMAP-36-induced OMVs (pOMVs) with heat-induced OMVs (hOMVs) and sOMVs regarding size, morphology, stability and lipid composition. Furthermore, we investigated the effect of OMVs on expression of cell-surface markers and cytokines by porcine bone marrow-derived M1 macrophages (pBMDM1). To modulate these immune responses, the immunomodulatory effect of PMAP-36 addition to isolated OMVs was also investigated.

## 2 Materials and methods

### 2.1 Peptide synthesis

PMAP-36 was synthesized by Fmoc-chemistry at China Peptides (CPC scientific, Sunnyvale, CA, USA). PMAP-36 was purified by reverse phase high-performance liquid chromatography to a purity of >95%. The sequence of the peptide is as follows: Ac-GRFRRLRKKTRKRLKKIGKVLKWIPPIVGSIPLGCG (Scheenstra et al., 2019).

### 2.2 Bacterial strains and growth conditions

A clinical isolate of *B. bronchiseptica* from pig (BB-P19) provided by the Veterinary Microbiological Diagnostic Center ((VMDC), Division of Infectious Diseases & Immunology, Faculty of Veterinary Medicine, Utrecht University) was used throughout this study. For lipidomic analysis, also the canine isolate BB-D09 (from VMDC) and a *pldA* mutant derivative of this strain were used. The strains were grown on Difco^TM^ Bordet-Gengou (BG) agar plates (Becton, Dickinson and Company, Sparks, USA), containing 1% glycerol (Merck, Darmstadt, Germany) and supplemented with 15% (v/v) defibrinated sheep blood (Oxoid Ltd, Basingstoke, Hampshire, UK). Liquid cultures were grown in Verwey medium (Verwey et al., 1949) (pH 7.4) containing 0.1% (w/v) starch from potato (S2004, Sigma-Aldrich, St. Louis, MO, USA), 0.02% (w/v) KCl, 0.05% (w/v) KH_2_PO_4_, 0.01% (w/v) MgCl_2_•6 H_2_O (all from Merck), 0.002% (w/v) nicotinic acid (Sigma-Aldrich), 1.4% (w/v) Bacto^TM^ casamino acids (Becton, Dickinson and Company), and 0.001% (w/v) L-glutathione reduced (Sigma-Aldrich).

### 2.3 OMV isolation

To obtain OMVs from *B. bronchiseptica*, bacteria were grown overnight to an OD_590_ of approximately 1.5. Before OMV isolation was initiated, bacteria were incubated for one hour either at 56°C or with 0.5 μM of PMAP-36 at 37°C. Subsequently, bacterial cells were removed by centrifugation for 30 min at 4700 x g. The supernatant was passed through a 0.45 μm Whatman filter (GE Healthcare, Chicago, Illinois, USA) and centrifuged at 40,000 rpm for 2 h at 4°C (Ti-70 rotor, Beckman coulter, Brea, California, USA). The supernatant was decanted, and the transparent pellet was dissolved in 2 mM Tris-HCl (pH 7.5, Sigma-Aldrich) in a volume corresponding to 2% of the bacterial culture.

### 2.4 Sodium dodecyl sulfate–polyacrylamide gel electrophoresis (SDS-PAGE)

Acrylamide gels (14%) were prepared as previously described (Biorad, General tips for handcasting 2014). For localization studies, samples were first treated with proteinase K (50 µg/mL, Thermo Fisher Scientific, Osterode am Harz, Germany) for 1 h at 37°C and then with phenylmethylsulfonyl fluoride (PMSF, Sigma-Aldrich) for 30 min at room temperature. For analysis of protein content, OMVs were diluted in 2x concentrated sample buffer containing 5% v/v β-mercaptoethanol (Sigma-Aldrich), incubated for 10 min at 95°C, and 20 μL were loaded on gel. Gels were run for 30 min at 50 V and then another 60 min at 150 V. Gels were stained with 0.1% (w/v) Coomassie Brilliant Blue R-250 (Serva, Heidelberg, Germany) in 50:40:10 UltraPure water (MQ): methanol: acetic acid (Sigma-Aldrich, Honeywell, Charlotte, North Carolina, USA) and destained overnight in 80:10:10 MQ: methanol: acetic acid. For staining LPS with silver (Tsai and Frasch, 1982), samples were diluted in 2x sample buffer as described above. Subsequently, samples were treated with proteinase K (50 μg/mL) for 1 h at 60°C, and 12 μL were loaded on gel. After running, gels were rinsed with MQ for 5 min. Next, gels were fixed for 1 h with 40:10:50 isopropanol: acetic acid: MQ (Honeywell and Sigma-Aldrich) and oxidized with a fresh solution of 0.7% periodic acid (Sigma-Aldrich) for 10 min. The gel was then rinsed four times with MQ for 15 min and stained with 20% AgNO_3_ (Merck) in 0.1 M NaOH and 0.0025% ammonia (both from Sigma-Aldrich). The gel was rinsed a further three times with MQ and developed for approximately 2 min with 0.005% (w/v) citric acid (Sigma-Aldrich) in 0.000185% (v/v) formaldehyde (Sigma-Aldrich). Gels were imaged with a Universal Hood III (Biorad, Hercules, California, USA).

### 2.5 Bicinchoninic acid (BCA) assay

Total protein concentration of isolated OMVs was determined using the Pierce BCA assay (Thermo Fisher Scientific). All samples were corrected for the signal of Verwey medium which was taken along during OMV isolation. In short, 25 μL of sample, supplemented with 2% SDS (Invitrogen, Carlsbad, California, USA), were incubated with 200 μL of working reagent at 37°C for 2 h. Absorbance was measured at 562 nm with FLUOstar Omega (BMG Labtech, Ortenberg, Germany). Bovine serum albumin (BSA, Sigma-Aldrich) was used as reference.

### 2.6 FM4-64 assay

Total lipid concentration of isolated OMVs was determined using the membrane-inserting fluorescent dye FM4-64 (Invitrogen). Samples (25 μL) were incubated with 200 μL FM4-64 (2.25 μg/mL) at 37°C for 10 min. Samples were excited at 485 nm and fluorescence was measured at 670 nm with the FLUOstar Omega.

### 2.7 Dynamic light scattering (DLS)

Samples for DLS were diluted 10-fold in 2 mM Tris-HCl unless stated otherwise. Samples were measured in micro-volume cuvettes (Sarstedt, Nümbrecht, Germany) on a Zetasizer nano (Malvern Panalytical, Malvern, UK) with a scatter angle of 173°. The standard polystyrene latex was used with a refractive index of 1.590 and absorbance of 0.010. Water was used as solvent (viscosity of 0.8872, refractive index of 1.330). Three measurements of 10-100 samplings were performed at 25°C unless stated otherwise. For the temperature gradient, steps of 5°C from 25-50°C were measured. Samples were equilibrated for 2 min and measured for 5 min.

### 2.8 Lipidomics

OMV pellets were obtained as described above but dissolved in PBS instead of Tris-HCl. Lipids from OMVs were extracted using the method described by Bligh and Dyer (Bligh and Dyer, 1959). Lipid extracts were dried under N_2_, dissolved in 100 μL of chloroform and methanol (1:1), and injected (10 μL) into a hydrophilic interaction liquid chromatography column (2.6 μm HILIC 100 Å, 50 × 4.6 mm, Phenomenex, CA). Lipid classes were separated by gradient elution on an Infinity II 1290 UPLC (Agilent, CA) at a flow rate of 1 mL/min. A mixture of acetonitrile and acetone (9:1, v/v) was used as solvent A, while solvent B consisted of a mixture of acetonitrile, H_2_O (7:3, v/v) with 50 mM ammonium formate. Both A and B contained 0.1% formic acid (v/v). Gradient elution was done as follows (time in min, % B): (0, 0), (1, 50), (3, 50), (3.1, 100), (4, 100). No re-equilibration of the column was necessary between successive samples. The column effluent was connected to a heated electrospray ionization source of an Orbitrap Fusion mass spectrometer (Thermo Scientific, MA) operated at -3600 V in the negative ionization mode. The vaporizer and ion transfer tube were set at a temperature of 275°C and 380°C, respectively. Full scan measurements (MS1) in the mass range from 450 to 1150 amu were collected at a resolution of 120.000. Parallelized data-dependent MS2 experiments were done with HCD fragmentation set at 30 V, using the dual-stage linear ion trap to generate up to 30 spectra per second. Data processing was based on the package ‘XCMS’ for peak recognition and integration (Smith et al., 2006). Lipid classes were identified based on retention time and molecular species were then matched against an *in silico* generated lipid database. This database was constructed based on observed and theoretical fatty acyls and phospholipids, as well as theoretical ornithine lipids and is available at http://www.lipidomics.nl/resources. Mass accuracy of annotated lipids was typically below 2 ppm.

### 2.9 Electron microscopy (EM)

For negative staining of OMVs, a protocol was provided by the Cell Microscopy Center (CMC, University Medical Center, Utrecht). In short, copper grids were carbon activated, incubated with 10 μL vesicle solution for 10-30 min and washed three times with phosphate-buffered saline (PBS, Thermo Fisher Scientific). The solution was fixed on the grids using 1% glutaraldehyde (Sigma-Aldrich) in PBS for 10 min and washed two times with PBS and subsequently four times with MQ. The grids were then briefly rinsed with methylcellulose/uranyl acetate (pH 4, provided by the CMC) and incubated for 5 min with methylcellulose/uranyl acetate (pH 4) on ice. Grids were looped out of the solution and air dried. Samples were imaged on a Tecnai-12 electron microscope (FEI, Hillsboro, Oregon, USA).

### 2.10 Porcine primary macrophages

Primary cells isolated from pig bone marrow were differentiated into M1 macrophages using porcine granulocyte-macrophage colony-stimulating factor (GM-CSF) as previously described (Gao et al., 2018). In short, the bone marrow cells were thawed, seeded at 50,000 cells per well, and cultured in 0.1% GM-CSF (Biorad) in RPMI +/+ (Thermo Fisher Scientific). At day 3 of the culture, cells were supplemented with 50 μL of 0.1% GM-CSF. At day 6, the differentiated macrophages were stimulated for 24 h with isolated OMVs (0.5 µg/mL protein) or purified LPS (10 ng/mL, kindly provided by J. Pérez Ortega, Utrecht University). Thereafter, cell markers and cytokines were measured using fluorescence-activated cell sorting (FACS, Becton, Dickinson and Company) and enzyme-linked immunosorbent assay (ELISA), respectively. For FACS, antibodies against the following surface markers and their dilutions were: Swc3α-PE (1:4000, Invitrogen, Carlsbad, California, USA), recombinant CTLA-4-MuIg-APC (1:1000, Ancell, Stillwater, Minnesota, USA) which binds to porcine CD80/86, CD163-FITC (1:1000), and human CD14-PB (1:100, both from Biorad). For cytokine detection, corresponding ELISA kits were used (R&D Systems, Minneapolis, Minnesota, USA).

## 3 Results

### 3.1 Dose determination of PMAP-36 treatment

**Fig. 1:**
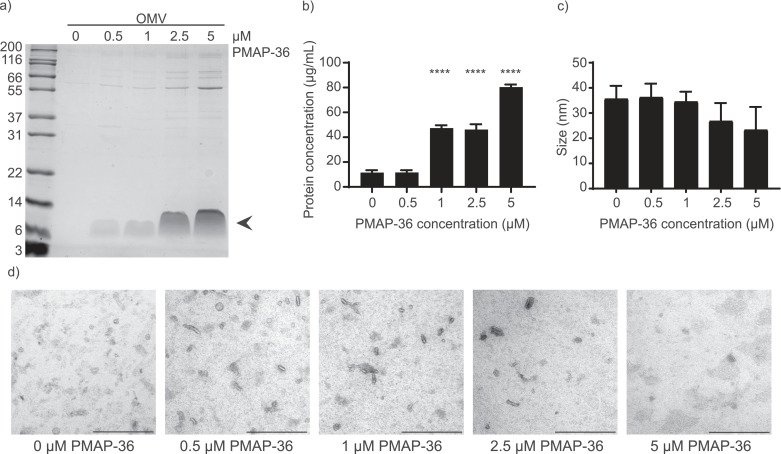
**Effect of PMAP-36 on OMV release of B. bronchiseptica**. (a) Increasing concentrations of PMAP-36 were supplemented during OMV induction and isolated OMVs were analyzed with SDS-PAGE. Black arrow points to PMAP-36 dimers present in isolated OMVs. (b) Protein concentrations of isolated OMVs were quantified using the BCA assay. Shown is the mean of three measurements with SD. Significant differences are indicated by ****p<0.0001, obtained using a one-way ANOVA with a post-hoc Dunnett test, compared to 0 µM PMAP-36. (c) Size of isolated OMVs was determined using DLS. Shown is the mean of three measurements with SD. (d) Isolated OMVs were visually inspected using EM. Bars represent 200 nm. Shown is a representative image of three individual experiments.

To determine whether PMAP-36 stimulates OMV release, *B. bronchiseptica* strain BB-P19 was incubated with PMAP-36 at concentrations varying from 0-5 µM. The maximum concentration tested, 5 μM, is 4-fold below the minimum bactericidal concentration (MBC) for an overnight culture (data not shown). First, OMV release was studied by analyzing the protein content of isolated OMV fractions using SDS-PAGE, which revealed an increase in protein concentration with increasing PMAP-36 concentrations (Fig. 1a). Remarkably, PMAP-36 was observed in the isolated OMV fractions around 7 kDa, presumably representing the dimeric form of the peptide despite the presence of β-mercaptoethanol in the sample buffer. An increase in protein concentration in the isolated OMV fraction was confirmed quantitatively using a BCA assay (Fig. 1b). An increase in protein concentration with increasing PMAP-36 concentrations suggests an increase in OMV release. However, at higher concentrations, the mere increase of PMAP-36 present in the OMVs could also be reflected in a higher protein signal in the BCA assay. Furthermore, a higher protein concentration does not necessarily correspond to an increase in intact OMVs, but can also be caused by soluble proteins. Therefore, the integrity of the released OMVs was investigated by EM visualization and size measurements by DLS. It was observed that at the highest concentrations of PMAP-36, the average size of OMVs tended to decrease (Fig. 1c). This result could indicate that OMVs are disintegrating at high PMAP-36 concentrations. The electron micrographs also suggested that less OMVs were present in the samples at 2.5 and 5 μM PMAP-36, probably due to disintegration of OMVs. Furthermore, gray patches were observed in the background at 5 μM of PMAP-36, indicating protein from the sample had dried and was stained (Fig. 1d). This result suggests that OMVs are disrupted at high PMAP-36 concentrations and release their protein content. Furthermore, DLS and EM showed that 1 µM of PMAP-36 was the maximum safe concentration to induce OMVs. Therefore, to prevent any OMV disruption, a safe concentration of 0.5 μM of PMAP-36 was used in further experiments.

To investigate the localization of PMAP-36, inside or outside the OMV, proteinase K digestion was applied followed by analysis of the protein patterns using SDS-PAGE. The PMAP-36 band of around 7 kDa was lost in the OMV samples, indicating PMAP-36 was accessible for proteinase K (Fig. S1). This suggests that PMAP-36 is on the outside of the OMV. Remarkably, PMAP-36 digestion was incomplete in the control condition, i.e. when pure peptide was digested with proteinase K. This could be due to aggregation, therefore rendering PMAP-36 less accessible for digestion.

### 3.2 Protein quantification of isolated OMVs

**Fig. 2:**
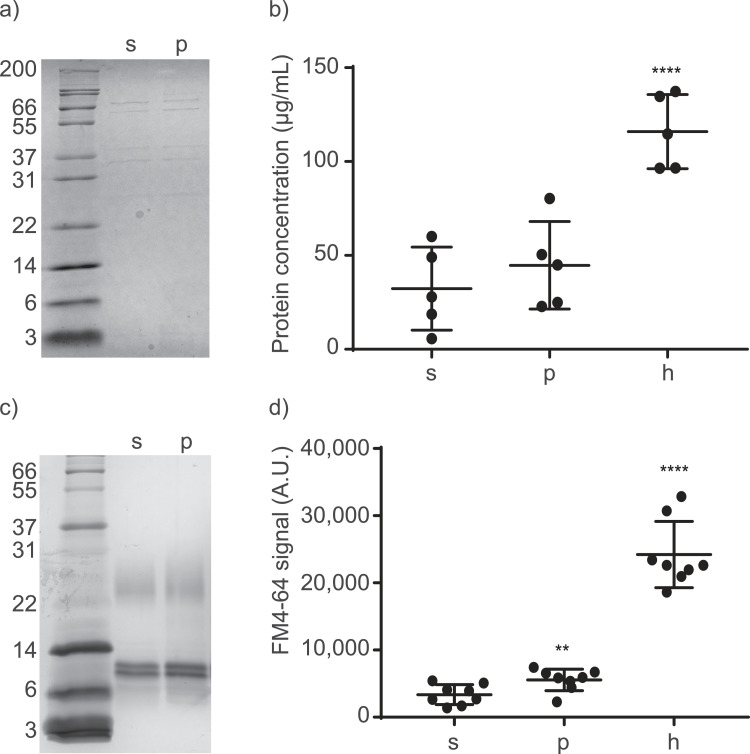
**Protein and lipid quantification of induced OMVs**. (a-b) Protein content and concentrations of isolated OMVs were determined using (a) SDS-PAGE and quantified using (b) BCA assay (n=5). (c) LPS content of isolated OMVs was visualized using silver staining on SDS-PAGE and (d) total lipid concentration was quantified using FM4-64 lipid dye (n=8). s = sOMVs, p = pOMVs, h = hOMVs. Significant differences compared to sOMVs are indicated by **p<0.01, ****p<0.0001, obtained using a one-way ANOVA with a post-hoc Dunnett test. Gels are representative of three individual experiments.

The isolated vesicles were inspected using SDS-PAGE to visualize differences in OMV release. Protein patterns on SDS-PAGE were identical for sOMVs and pOMVs (Fig. 2a). Visually, no differences in quantity could be observed. Therefore, the protein concentration in the isolated OMVs was quantified using a BCA assay. Since heat-treatment was previously shown to induce OMVs in *B. pertussis* and in a canine strain of *B. bronchiseptica* (accompanying manuscript by E. F. de Jonge), it was taken along as control treatment. PMAP-36 treatment showed a slight, but not significant, increase in the protein concentration of isolated OMVs (1.4x), whereas heat treatment did show a significant increase in protein concentration of isolated OMVs (3.6x) (Fig. 2b).

### 3.3 Lipid quantification of isolated OMVs

In addition to protein quantification, OMV release was also determined by analysis of lipid quantities. LPS was visualized by staining with silver (Fig. 2c). Two bands for the lipid A plus core sugar moiety were visible around 10 kDa, with the higher band caused by PagP activity, which has been shown to transfer a 16 carbon acyl chain from phospholipids to *B. bronchiseptica* LPS (Preston et al., 2003). Also O-antigen-containing LPS was observed around 25 kDa (Peppler, 1984). A slight increase in LPS quantity was observed for pOMVs. Subsequently, lipid concentrations were compared using the FM4-64 lipid dye, revealing a significant 1.7-fold increase for pOMVs and a 6.5-fold increase for hOMVs relative to sOMVs (Fig. 2d).

### 3.4 Morphological characterization of isolated OMVs

**Fig. 3:**
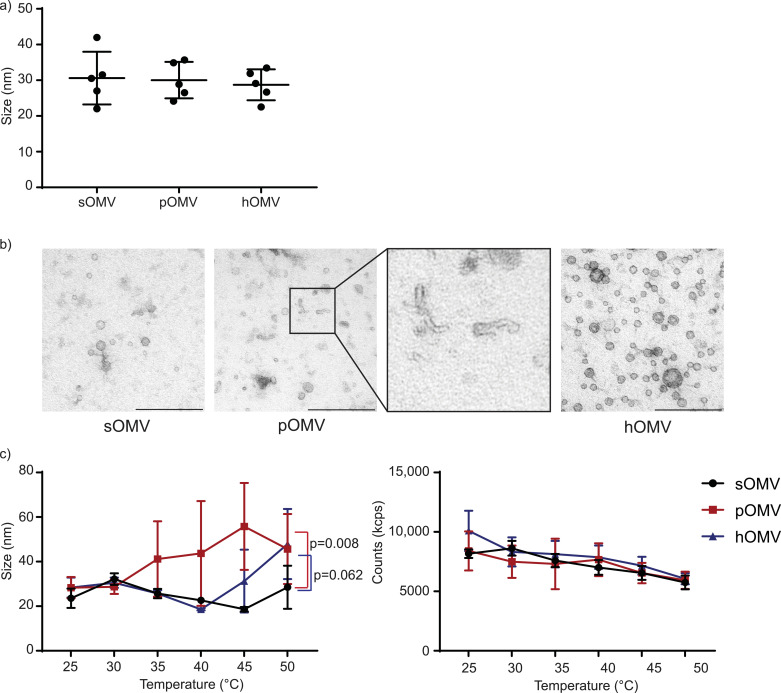
**Morphology and stability of induced OMVs**. (a) Size of isolated OMVs was measured with DLS (n=5). (b) Isolated OMVs visualized using EM. Insert of pOMVs is four times enlarged. Shown is a representative image of three individual experiments. Bars represent 200 nm. (c) Stability of OMVs was assessed by size (left panel) and count (right panel) variations over a temperature gradient (n=3). Shown is the mean with SEM. Statistic differences compared to sOMVs were calculated using a linear mixed-model analysis with a post-hoc Dunnett test.

Previously, OMVs have been described as 20-300 nm spherical blebs of the OM (McBroom and Kuehn, 2007, Kulp and Kuehn, 2010). In this study, *B. bronchiseptica* OMVs were observed to be at the small end of this spectrum. The size of OMVs, measured with DLS, ranged from 20-40 nm (Fig. 3a). EM analysis revealed even smaller OMVs than those shown by DLS measurements, with vesicles as small as 15 nm (Fig. 3b). Neither PMAP-36 nor heat treatment affected OMV size. However, in pOMV samples, tubular structures were observed with EM (insert Fig. 3b). Presumably, PMAP-36 interacts with the OMV membrane and affects their shape.

### 3.5 Thermal stability of isolated OMVs

To determine the thermal stability of isolated OMVs, the size and counts of OMVs were measured with DLS while being subjected to a temperature gradient. Temperature was raised from 25°C to 50°C with 5°C steps and the OMVs from different induction methods were compared. sOMVs showed no differences in size with increasing temperature, suggesting they are stable at higher temperatures (Fig. 3c, left panel). However, pOMVs increased in size from 35°C onwards, indicating their aggregation or fusion. hOMVs tended to increase in size from 45°C onwards, being more stable than pOMVs, but slightly less stable than sOMVs. Counts decreased for all OMV types, likely due to sedimentation during the measurement (Fig. 3c, right panel).

### 3.6 Lipidomic analysis of isolated OMVs

**Fig. 4:**
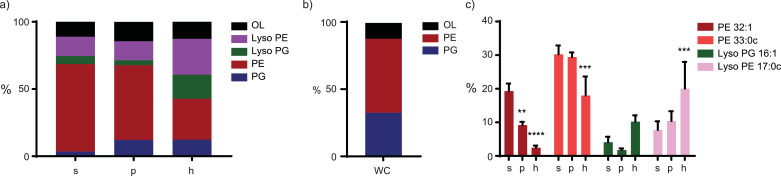
**Lipidomic analysis of isolated OMVs**. Isolated OMVs were subjected to mass spectrometry to determine lipid composition (n=3). (a) Total lipid composition of OMVs and (b) of whole cells. (c) Four most affected lipid species. s = sOMVs, p = pOMVs, h = hOMVs, WC = Whole Cells. Significant differences compared to sOMVs are indicated by **p<0.01, ***p<0.001, ****p<0.0001, obtained using a paired two-way ANOVA with a post-hoc Dunnett test.

The lipid classes in isolated OMVs were investigated to explain changes in morphology and stability. Differences in phospholipid composition of the different OMV preparations were clearly observed by mass spectrometric analysis. Cardiolipin was detected in the OMVs but was excluded from further analysis as it could not be reliably quantified due to interfering compounds in the OMV isolates. Of the phospholipid classes, phosphatidylglycerol (PG) was increased in OMVs obtained after treatment of bacteria with either PMAP-36 or heat (Fig. 4a). Furthermore, the lysophospholipid content in OMVs was increased after heat treatment, while the phosphatidylethanolamine (PE) content was reduced, consistent with its conversion into lyso-PE (Fig. 4a). When compared to the lipid composition of whole cells (WC), lysophospholipids appeared exclusively present in OMVs (Fig. 4b). The increase in lysophospholipids in hOMVs could be due to enzymatic hydrolysis by outer-membrane phospholipase A (OMPLA), which is encoded by the *pldA* gene. To test this possibility, we analyzed lipid species of OMVs isolated from a *pldA* mutant in a canine strain of *B. bronchiseptica*, BB-D09, since we were unable to introduce the mutation into BB-P19. The OMVs of the wild-type canine strain showed differences in lipid composition relative to those of the porcine strain, such as the relatively higher abundance of PG (Fig. S2). However, it was still considered a good model to investigate the influence of OMPLA. The *pldA* mutant was also subjected to heat or peptide treatment before OMV isolation and the lipidomic analysis of the OMVs indicated that although lysophospholipids were still detectable (Fig. S2), their abundance is strongly diminished in the *pldA* mutant. These data indicate that the majority of the lysophospholipids produced upon heat treatment results from OMPLA activity. The remaining portion of lysophospholipids, present in both sOMVs and hOMVs, might be the result of enzymes such as the acyltransferase PagP (Preston et al., 2003, Hwang et al., 2002).

Around 90% of the lipids could be identified as belonging to only thirteen lipid species (Fig. S3). PE was found to be the most prominent lipid class, specifically with 16:0, 16:1 or 17:0c fatty acid chains, where the ‘c’ indicates a cyclopropane moiety in the acyl chain. Concordantly, lysoPE 16:0, 16:1 and 17:0c were the most prominent lysophospholipids. *B. bronchiseptica* was shown before to contain mostly 16:0 and 17:0c fatty acid chains, but 16:1 appeared to be less prominent in previous datasets (Kawai and Moribayashi, 1982, Dees et al., 1980). In our study, 16:0 and 16:1 seem to be present in comparable amounts. The most affected lipid species in OMVs obtained after either peptide or heat treatment were PE 32:1, PE 33:0c, lysoPG 16:1 and lysoPE 17:0c (Fig. 4c). PE 33:0c was found to decrease only upon heat treatment, while PE 32:1 decreased upon both PMAP-36 and heat treatment. Furthermore, lysoPE 17:0c was found to increase in hOMVs. Presumably, lysoPE 17:0c is the product of enzymatic conversion of its diacyl counterpart, for instance by the acyltransferase activity of PagP. Additionally, lysoPG 16:1 increased in the hOMVs and showed a decreasing trend in pOMVs (Fig. 4c).

Next to phospholipids, another class of polar lipids was identified in the lipid extract. Ultrahigh resolution, accurate mass tandem mass spectrometry demonstrated notable amounts of ornithine lipids (OL) in OMVs (Fig. S4). This lipid class has previously been described to be present in Bordetella pertussis (Kawai and Moribayashi, 1982). Furthermore, this lipid contains two fatty acids that can vary, mostly being two 16:0 acyl chains (approximately 8-10%) or one 16:0 and one 14:0 (approximately 2-4%) (Fig. S3). Strikingly, the relative amounts of these lipids in the OMVs were not influenced by either treatment.

### 3.7 Immune response of pBMDM1 on OMVs induced by different methods

**Fig. 5:**
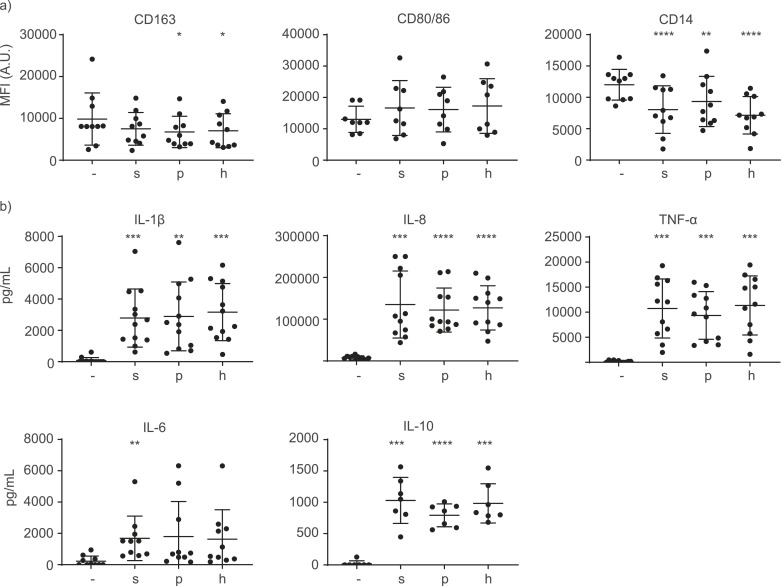
**pBMDM1 response to induced OMVs**. pBMDM1 macrophages were stimulated with isolated OMVs and activation was measured by determining (a) cell-surface markers using FACS and (b) cytokines using ELISA. MFI = mean fluorescence intensity. - = no stimulation, s = sOMVs, p = pOMVs, h = hOMVs (n=7-12). Significant differences compared to the non-stimulated control are indicated by *p<0.05, **p<0.01, ***p<0.001, ****p<0.0001, obtained using a repeated measures one-way ANOVA with Geisser-Greenhouse correction and post-hoc Dunnett test.

pBMDM1 were used to investigate immune responses induced by OMVs. After 24 h stimulation with OMVs, cells were gated on the myeloid marker SWC3α and pBMDM1 activation was determined by measuring cell-surface markers CD163, CD80/86 and CD14 using FACS. The expression of CD163 and CD80/86 was marginally affected after stimulation of pBMDM1s with OMVs, whilst CD14 expression was significantly decreased (Fig. 5a). However, expression of all markers was similar after stimulation with sOMVs, pOMVs or hOMVs. Macrophage activation was further determined by measuring the release of cytokines TNF-α, IL-1β, IL-6, IL-8 and IL-10 using ELISA. Cytokine release was induced by OMVs but again no differences were observed between different OMVs (Fig. 5b). These results suggest that although all OMVs could potently induce immune responses, these were not affected by the induction method used for OMV release.

### 3.8 Immune response of pBMDM1 to OMVs supplemented with PMAP-36

**Fig. 6:**
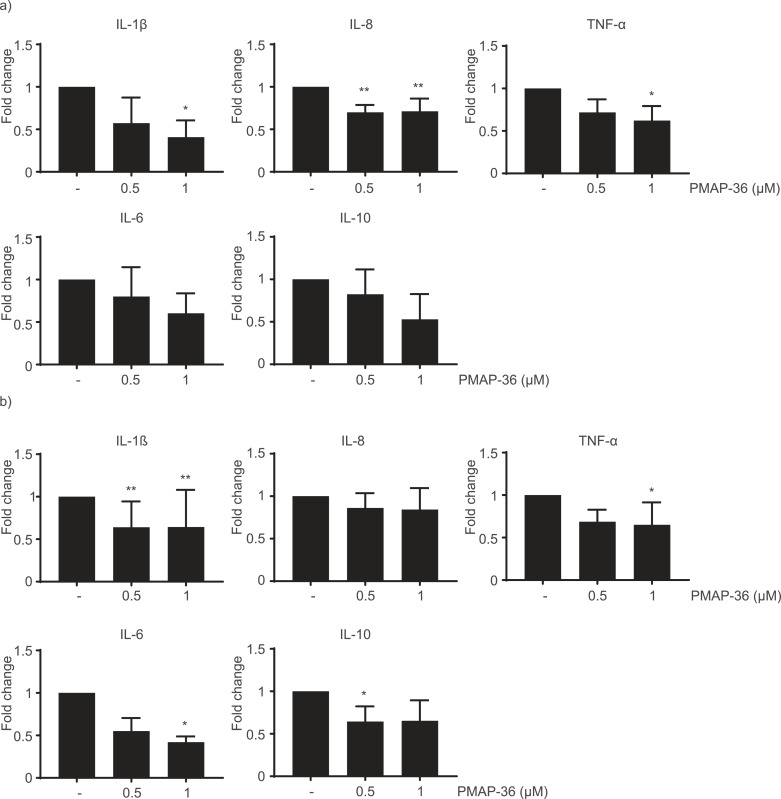
**PMAP-36 modulation of pBMDM1 cytokine expression in response to OMVs**. pBMDM1 macrophages were stimulated with (a) sOMVs or (b) hOMVs in the presence of different concentrations of PMAP-36. Cytokine secretion was determined by ELISA. Values were converted to fold change with respect to the results obtained with OMVs not supplemented with PMAP-36. Shown is the mean with SD. Significant differences are indicated by *p<0.05, **p<0.01, obtained by using a linear mixed-model analysis on the raw data with a post-hoc Dunnett test (n=3-7).

The lack of effect of PMAP-36 on immune responses in peptide-induced OMVs could be explained by possible inaccessibility or a too low concentration of PMAP-36 in OMVs after isolation to neutralize LPS. Therefore, free PMAP-36 was added to isolated OMVs and the subsequent immunomodulation of pBMDM1 responses was studied. Expression of cell-surface markers CD163 and CD80/86 showed no differences upon stimulation with OMVs and PMAP-36, whilst CD14 slightly increased but only significantly for stimulation with hOMVs and PMAP-36 (Fig. S5), indicating neutralization of the LPS-induced CD14 decrease observed earlier. Furthermore, cytokine release showed a decrease in pro-inflammatory IL-1β, IL-6, IL-8 and TNF-α, as well as in anti-inflammatory IL-10 secretion upon PMAP-36 addition to OMVs (Fig. 6).

PMAP-36 has previously been shown to interact with *E. coli* LPS and neutralize subsequent macrophage responses (Coorens et al., 2017). To investigate whether this mechanism holds true for PMAP-36 and *B. bronchiseptica* LPS, experiments were performed using purified *B. bronchiseptica* LPS. Cell-surface marker expression was not affected by addition of PMAP-36. However, addition of PMAP-36 did result in a decreasing trend in LPS-induced cytokine release of pBMDM1 (Fig. S6). The decrease was significant for TNF-α and IL-10, suggesting that the immune suppression of PMAP-36 was inhibiting macrophage activation in general, since both pro- and anti-inflammatory cytokine expression was decreased by PMAP-36.

## 4 Discussion

In this study, a sub-lethal concentration of PMAP-36 was applied to *B. bronchiseptica* to induce OMV release. The pOMVs were extensively assessed to investigate their properties and qualities. Lipid quantification showed that PMAP-36 treatment significantly increased OMV release (Fig. 2d). This increase is consistent with the literature, which shows that stress induces release of OMVs (von Bergen et al., 2012, Eberlein et al., 2018, Schwechheimer and Kuehn, 2013, Chan et al., 2017, McBroom and Kuehn, 2007, Kulkarni et al., 2015) (accompanying manuscript by E. F. de Jonge). Previously, it has been demonstrated that antibiotics increased OMV release in *E. coli*. However, the effect varied from a 2- to 4-fold induction for tetracycline, ampicillin, and ceftriaxone, up to a 10-fold induction by polymyxin B (Manning and Kuehn, 2011). Additionally, in *Pseudomonas aeruginosa*, treatment with D-cycloserine and polymyxin B both demonstrated a 6 to 9-fold increase in OMV release (MacDonald and Kuehna, 2013). The stress induction by PMAP-36 might be explained by its interactions with membranes. Cationic HDPs interact electrostatically with the negatively charged bacterial membrane. In *B. bronchiseptica* however, the phosphate groups on LPS appeared to be shielded to a large extent by glucosamines (manuscript in preparation by J. Pérez Ortega), possibly explaining the small effect of PMAP-36 on OMV formation. Additionally, because HDPs contain a hydrophobic region, the hydrophobic core of the membrane can be reached and high concentrations of HDPs can lead to membrane permeabilization, for which several models have been suggested (Mookherjee et al., 2020, Zhang and Gallo, 2019). Before HDP-induced membrane permeabilization occurs, OMVs could already be formed due to the bulging effect of HDPs on the membrane. OMV release in response to HDPs could be a defense mechanism, with bacteria either using OMVs to remove HDP-affected membranes or using them as decoy membrane for the HDPs (Manning and Kuehn, 2011).

PMAP-36 indeed interacted with the bacterial membrane, since it was found in the OMV fraction. Proteinase K digestion revealed PMAP-36 to be, at least partially, on the outside of the OMV (Fig. S1). Remarkably, PMAP-36 digestion by proteinase K in the control condition, i.e. pure PMAP-36 without OMVs, was incomplete. A possible explanation is that PMAP-36 in pure solution aggregates, rendering large parts of the peptide inaccessible for proteinase K, while the peptide on OMVs is prevented from aggregation, rendering the peptide accessible. This suggests that PMAP-36 is not deeply inserted into the OMV membrane. PMAP-36 induction showed an increasing trend for protein concentration and a significant increase in lipid quantities in the isolated OMV fraction, suggesting PMAP-36 treatment enhances OMV release (Fig. 2). It needs to be taken into account that both assays could be influenced by the presence of PMAP-36 in the OMVs. PMAP-36 gives a signal in the BCA assay, being partly responsible for the increase in protein concentration. Furthermore, the presence of PMAP-36 could potentially hamper or promote insertion of FM4-64 into the OMV membrane, influencing the quantification. In any case, heat treatment was observed to increase OMV release to a larger extent than PMAP-36 treatment, making it more suitable method for production of large amounts of OMVs (accompanying manuscript by E. F. de Jonge). However, stress might alter OMV properties, and stress-induced OMVs must therefore be characterized further to confirm their potential for vaccine applications.

Occasionally, pOMVs appeared cylindrical in EM studies (Fig. 3). The morphology of synthetic vesicles has previously been described to be influenced by peptides (Polido et al., 2019). The use of PMAP-36 for OMV release also affected the stability of resulting OMVs. OMVs induced by PMAP-36 stress started to aggregate around 35°C. Similarly, hOMVs tended to aggregate around 45°C, but size differences were not significant. In both cases, variation was considerable, probably due to the fact that not all OMVs aggregated at a similar temperature or time point. At 40°C, for example, part of the OMV population might still have their original size, while another part has already aggregated. To decrease heterogeneity, temperature incubations could be prolonged in time. However, sOMVs, pOMVs and hOMVs all showed a slight decrease in counts during the measurement, probably due to sedimentation, so this could influence the measurement if time would be too prolonged. Remarkably, counts of pOMVs and hOMVs did not decrease quicker than those of sOMVs, which one would expect when particles aggregate. Perhaps, larger particles are counted more frequently and therefore this was not observed. Since PMAP-36 was observed to be present in the isolated OMVs, it could be inserted and thereby destabilize the membrane. However, the stability of sOMVs to which 0.5 µM PMAP-36 was added did not decrease with increasing temperatures (data not shown). Furthermore, EM showed no morphological differences of the sOMVs after PMAP-36 addition (data not shown). Possibly, PMAP-36 is unable to insert at that concentration into OMVs after their isolation.

To further explain differences observed in stability, lipid species in isolated OMVs were identified using mass spectrometry (Fig. 4). sOMVs contained mostly PE (Fig. S3), in agreement with previous studies of the *B. bronchiseptica* total cell lipidome. These previous studies are extended by our lipidomic characterization of OMVs, demonstrating the presences of specifically PE 33:0c (30.2%) and PE 32:1 (19.2%). This is in agreement with the reported preference of *B. bronchiseptica* for 16:0, 16:1 and 17:0c fatty acids (Kawai and Moribayashi, 1982, Dees et al., 1980). PE is a lipid with a relatively small head group, preferring structures with a negative curvature, and it could make up the inner monolayer of the OMV. In pOMVs, the amount of PG species, which are negatively charged, was enriched relative to that in sOMVs. This can be linked to the interaction between negatively charged PG and positively charged PMAP-36. During OMV formation, phospholipid concentration in the outer leaflet increases (Roier et al., 2016). The bacteria could use negatively charged OMVs to dispose already bound PMAP-36 or to act as decoy for free PMAP-36. The PG species in the pOMVs had identical fatty acid composition compared to the most abundant PE species in sOMVs, in accordance with the preference of *B. bronchiseptica* for these acyl chains (Fig. S3). Upon heat treatment on the other hand, there was a relative increase in lysophospholipids at the cost of diacyl phospholipids. Both OMPLA and PagP are enzymes in the OM capable of enzymatic conversion of phospholipids. OMPLA functions primarily as phospholipase A1 and is capable of removing an acyl chain, but also has phospholipase A2 and 1-acyl- and 2-acyllysophospholipase activity (Horrevoets et al., 1989). PagP is an acyltransferase and not only specifically removes a 16:0 acyl chain from phospholipids, but transfers this acyl chain to LPS (Preston et al., 2003). The relative increase of lysoPE 17:0c can be linked to the relative decrease of PE 33:0c, where a 16:0 fatty acid is lost, probably due to PagP transferring this acyl chain to LPS. Remarkably, the decrease of PE 32:1 did not result in an increase in lysoPE 16:0 or lysoPE 16:1, as one would expect. This could be due the activity of OMPLA, potentially converting PE into mere acyl chains and a glycerophosphoryldiester. Furthermore, the lysoPG content of hOMVs was increased relative to that in sOMVs, while diacyl PG didn't decrease. It needs to be taken into account that a decrease of a specific lipid in the OMV lipidome can also be the result of retention of that lipid in the bacterial OM and vice versa. Lipidomic analysis of the OMVs of a canine *pldA* mutant strain revealed that OMPLA-mediated enzymatic hydrolysis was the main process responsible for hydrolysis upon heat treatment. However, some production of lysophospholipids was still observed at a lower temperature, even in the *pldA* mutant. The active site of both OMPLA and PagP is situated in the outer leaflet of the bilayer, leading to lysophospholipids to also end up in the outer leaflet of OMVs. Since lysophospholipids only contain a single fatty acid, they tend to form structures with a high positive curvature, such as micelles (Fuller and Rand, 2001). Therefore, they are very well suited to make up the outer leaflet of a bilayer. However, lysophospholipids could decrease OMV stability. Furthermore, interactions between OMVs and immune molecules or cells could be influenced by the presence of lysophospholipids, since they have detergent-like activities.

Despite the different properties of the differently induced OMVs, immune responses did not differ (Fig. 5). In previous studies, immune responses to *E. coli* LPS were shown to decrease by addition of PMAP-36, due to an interaction between the peptide and LPS (Coorens et al., 2017). This led to the hypothesis that LPS in pOMVs would also be neutralized by the peptide, resulting in a decreased immune response. However, this was not observed. The three OMV preparations showed no difference in macrophage activation, suggesting the available PMAP-36 in pOMVs is unable to neutralize LPS. A possible explanation is that PMAP-36 is trapped by the OMVs and incapable of interacting with released LPS to neutralize TLR4 responses. Accordingly, free PMAP-36 was able to decrease cytokine release of pBMDM1 stimulated with pure *B. bronchiseptica* LPS (Fig. S6). To investigate whether PMAP-36 is inaccessible in pOMVs, sOMVs were supplemented with increasing concentrations of PMAP-36 before stimulation of macrophages. Whereas stimulation with hOMVs alone decreased CD14 surface expression (Fig. 5a), addition of PMAP-36 to hOMVs was shown to negate this decrease (Fig. S5). Furthermore, addition of PMAP-36 decreased cytokine release of OMV-stimulated pBMDM1 (Fig. 6). This shows that PMAP-36 is able to neutralize LPS in OMVs and to decrease immune responses. An alternative explanation for the lack of modulation by PMAP-36 present in pOMVs is that the PMAP-36 concentration present in these OMVs is plainly too low, but this is rather impossible in our hands to detect and compare. Apart from PMAP-36, also another, synthetic peptide was previously shown to reduce OMV-induced immune responses (Pfalzgraff et al., 2019) suggesting that peptides could be promising immunomodulatory molecules in vesicle-based vaccines. Notably, a decreasing trend was observed for induction of both pro- and anti-inflammatory cytokines upon supplementation of OMVs with PMAP-36 (Fig. 6). This suggests that the immune suppression by PMAP-36 is not specifically reducing either pro- or anti-inflammatory processes. Higher concentrations of PMAP-36 appeared to be toxic to macrophages and could therefore, unfortunately, not be tested (data not shown). Investigation of the immune modulatory properties of a range of HDPs might identify a less toxic peptide, specifically modulating the immune response.

For the human cathelicidin LL-37, it is well known that it has multiple immunomodulatory functions, including the ability to neutralize LPS-induced macrophage activation (Scott et al., 2002, Lai and Gallo, 2009, Vandamme et al., 2012). Several other cathelicidins, such as CATH-2 and PMAP-36, have also been shown to reduce TNF-α release from LPS-stimulated stimulated macrophages (Coorens et al., 2017). Moreover, synthetically designed peptides have proven to be very promising immunomodulators. Innate Defense Regulator peptides (IDRs) were shown to induce chemokine secretion while simultaneously reducing LPS-induced cytokine secretion *in vitro* and, likewise, inflammation *in vivo* (Niyonsaba et al., 2013, Wuerth et al., 2017). However, these peptides decrease secretion of both pro- and anti-inflammatory cytokines, which might not be necessary for an optimal immune response. When a different balance is needed between pro- and anti-inflammatory responses, lowering both is not appropriate. Therefore, screening larger sets of HDPs for their immunomodulatory properties might reveal some that skew the balance between pro- and anti-inflammatory responses which could be useful for vaccine applications. In OMV-based vaccines for instance, only pro-inflammatory responses have to be decreased. While for *B. pertussis* OMV-based vaccines are already favorable compared to whole-cell vaccines with respect to reactogenicity (Raeven et al., 2016), LPS is still present, which might cause undesirable side effects. HDPs might be promising molecules to address this issue, since they specifically neutralize LPS. Currently, the supplementation of vaccines with HDPs has already shown successful results, in both a bacterial subunit vaccine and a viral vaccine (Garg et al., 2017). Some well-known HDPs, such as indolicidin, LL-37 and BMAP-27, as well as derivatives thereof were used to enhance pro-inflammatory properties (van den Hurk et al., 2016). This shows the potential of HDPs as excipient molecules and their ability to be adjusted to obtain optimal immune modulation.

## 5 Concluding remarks

We have investigated the effect of PMAP-36 on OMV release. OMV release was only slightly induced by bacterial stimulation with PMAP-36. Furthermore, we have investigated the subsequent immune response of isolated OMVs and the effect of PMAP-36 addition thereon. Innate immune responses to OMVs were effectively decreased after addition of PMAP-36. This indicates that HDPs are promising excipients for vaccine applications. Investigating the immunomodulatory properties of other HDPs might result in an ideal adjuvant for OMV-based vaccines.

## CRediT authorship contribution statement

**Melanie D. Balhuizen:** Conceptualization, Data curation, Formal analysis, Investigation, Methodology, Writing - original draft. **Chantal M. Versluis:** Investigation. **Roel M. van Harten:** Methodology. **Eline F. de Jonge:** Methodology, Resources. **Jos F. Brouwers:** Formal analysis. **Chris H.A. van de Lest:** Formal analysis. **Edwin J.A. Veldhuizen:** Supervision, Conceptualization, Writing - review & editing. **Jan Tommassen:** Writing - review & editing. **Henk P. Haagsman:** Supervision, Conceptualization, Funding acquisition, Writing - review & editing.

## Declaration of Competing Interest

This research was supported in part by NWO-TTW grant 14921 and 14924 to the Bac-Vactory program. Part of this work is included in a European patent application (EP20187477.3) with EFdJ, MDB, HPH, and JT as inventors.

## Appendix Supplementary materials

Image, application 1
