# Supplementary material for: PMAP-36 reduces the innate immune response induced by Bordetella bronchiseptica-derived outer membrane vesicles
Source: Curr Res Microb Sci. 2020 Sep 25;2:100010. doi: 10.1016/j.crmicr.2020.100010 (PMC8610334; doi:10.1016/j.crmicr.2020.100010)
Supplement: Supplementary file 1 [file mmc1.docx]

**Supplementary information**


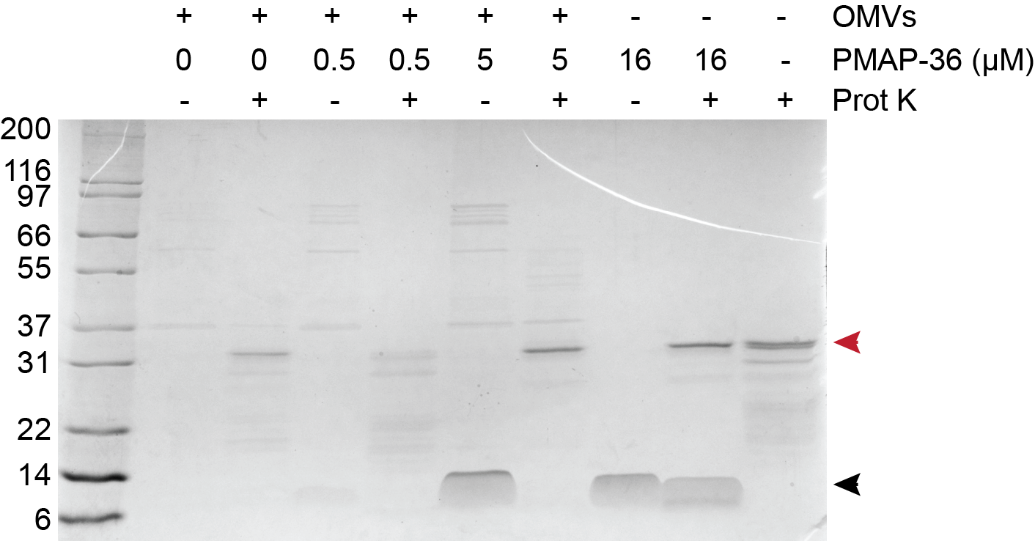


**Figure S1: Localization of PMAP-36 in the isolated OMVs.** pOMVs were subjected to proteinase K treatment and the integrity of PMAP-36 was assessed using SDS-PAGE. Red and black arrows indicate respectively proteinase K and PMAP-36 on the gel. The first six lanes contain pOMVs isolated with different PMAP-36 concentrations. The next two lanes contain pure PMAP-36 as control.


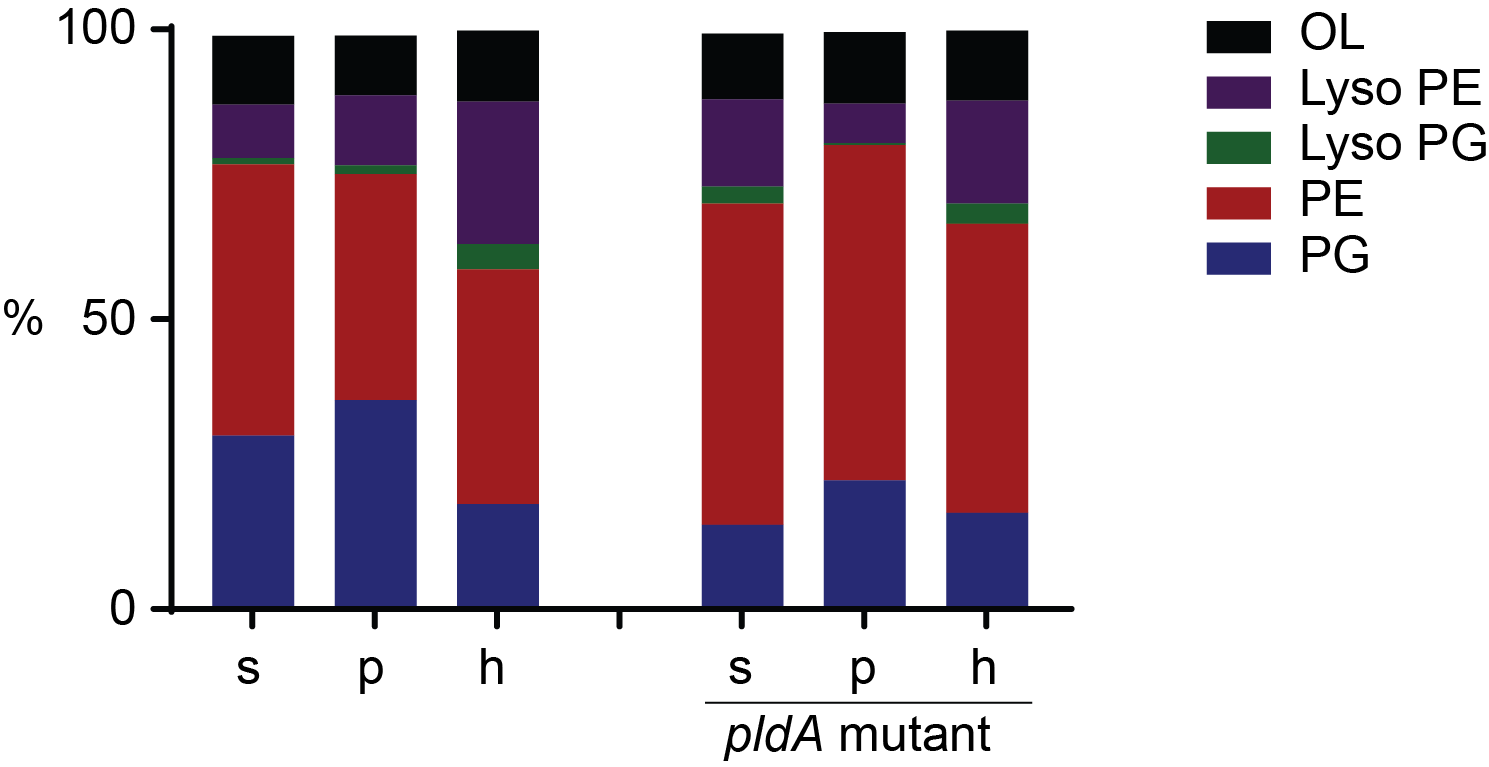


**Figure S2: Lipidomic analysis of OMVs produced by B. bronchiseptica strain BB-D09 and its pldA mutant derivative.** B. bronchiseptica was subjected to different treatments and phospholipid composition of isolated OMVs was determined using mass spectrometry (n=3). Cardiolipin was detected but could not be quantified. s = sOMVs, p = pOMVs, h = hOMVs.


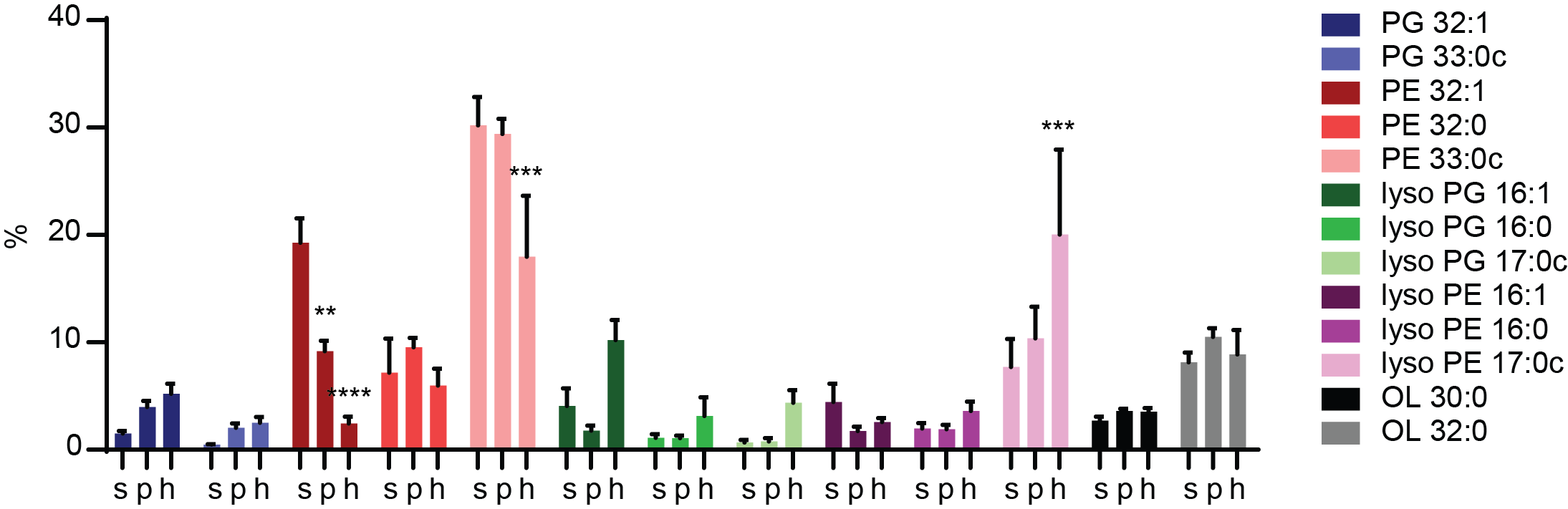


**Figure S3: Most prominent lipid species in B. bronchiseptica BB-P19 OMVs.** B. bronchiseptica was subjected to different treatments and phospholipid composition of OMVs was determined using mass spectrometry. Depicted are the phospholipid classes that account together for approximately 90% of the total phospholipids. s = sOMVs, p = pOMVs, h = hOMVs. Significant differences compared to sOMVs are indicated by **p<0.01, ***p<0.001, ****p<0.0001, obtained using a paired two-way ANOVA with a post-hoc Dunnett test.


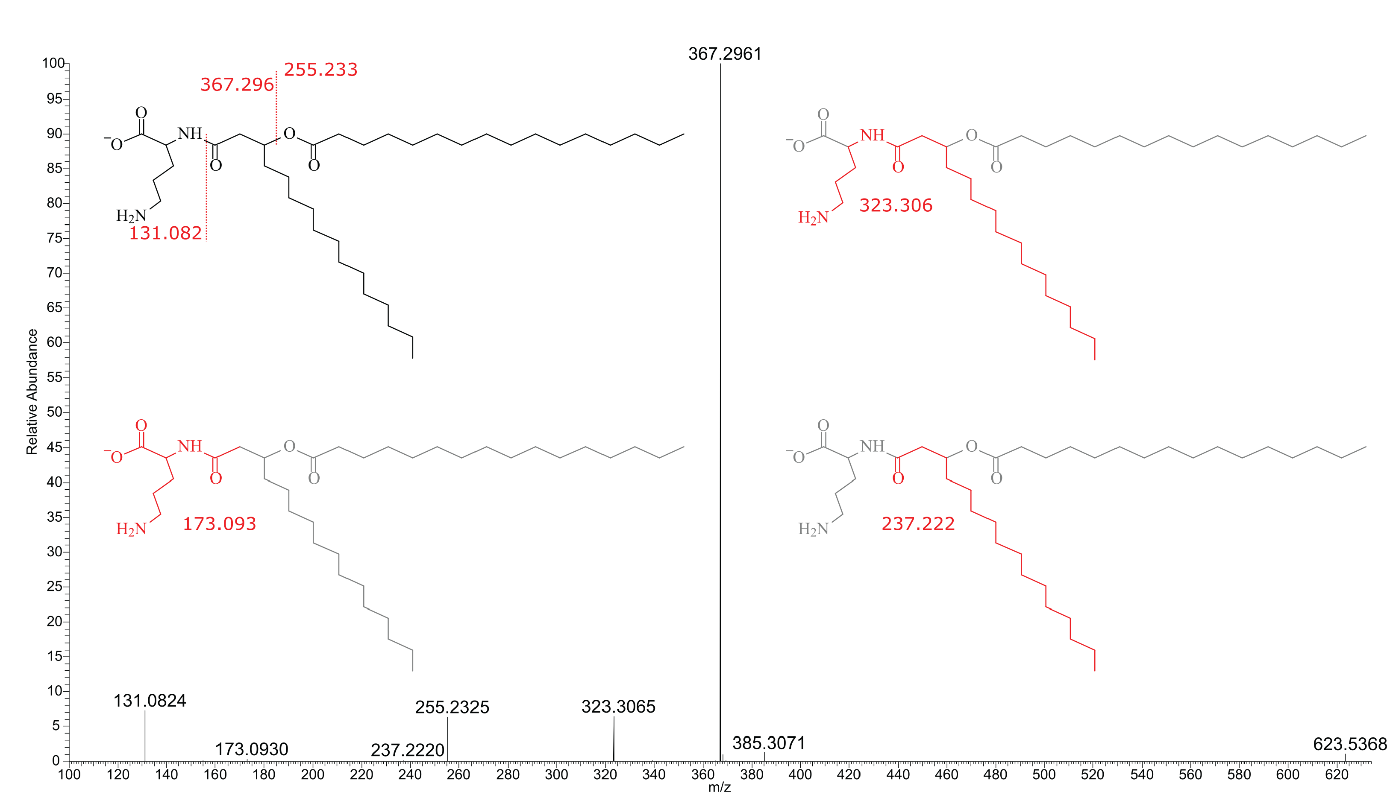


**Figure S4: Ultra-high resolution accurate mass MS2 spectrum demonstrating the presence of ornithine lipid in OMVs.** Structures matching m/z values are displayed in red, with the full structure displayed on the top left, corresponding to the precursor ion at m/z 623.5368.


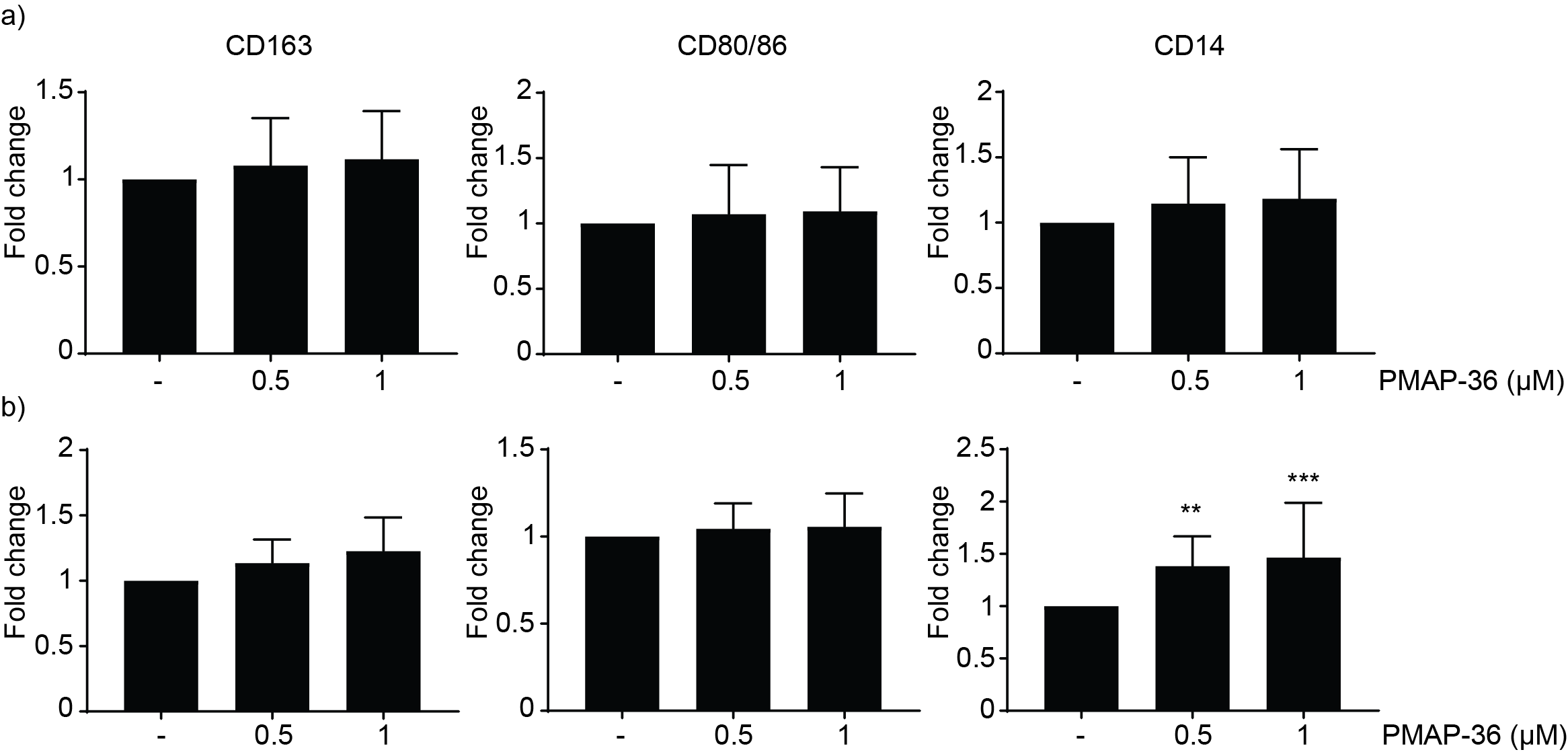


**Figure S5: PMAP-36 modulation of pBMDM1 cell-surface markers in response to OMVs.** pBMDM1 macrophages were stimulated with (a) sOMVs or (b) hOMVs in the presence of different concentrations of PMAP-36. Cell-surface markers CD163, CD80/86 and CD14 were measured using FACS. Values were converted to fold change compared to the values for OMVs without supplemented PMAP-36. Significant differences are indicated by *p<0.05, **p<0.01, ***p<0.001, obtained by using a linear mixed-model analysis on the raw data with a post-hoc Dunnett test (n=8).


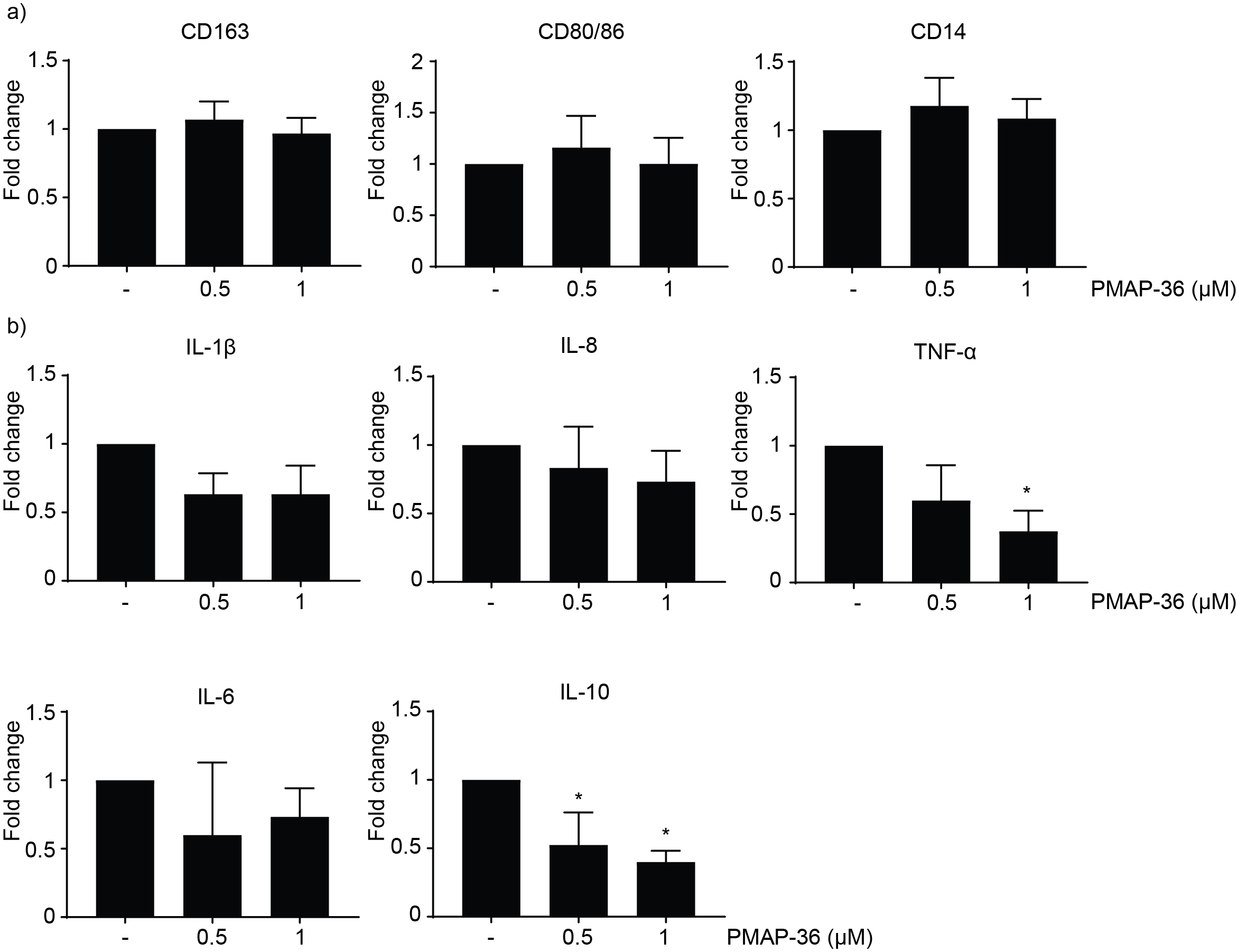


**Figure S6: PMAP-36 modulation of pBMDM1 response to LPS of B. bronchiseptica.** Porcine BMDM1 macrophages were stimulated with LPS isolated from B. bronchiseptica supplemented with different concentrations of PMAP-36. Activation was measured by (a) cell surface markers and (b) cytokines. Values were converted to fold changes compared to LPS without supplemented PMAP-36. Significant differences are indicated by *p<0.05, obtained by using a linear mixed-model analysis on the raw data with a post-hoc Dunnett test (n=3-6).
